# Supplementary material for: Are carbon emissions trading and green financial instruments synergistic? -Comprehensive quantitative research based on content analysis
Source: PLoS One. 2024 Mar 7;19(3):e0298601. doi: 10.1371/journal.pone.0298601 (PMC10919601; doi:10.1371/journal.pone.0298601)
Supplement: S1 Data — (ZIP) [file pone.0298601.s001.zip › Data/地级市Z_F工作报告.zip!/╣π╢1⁄2/╗▌╓▌╩╨/└┤╫╘╣1⁄2╓┌║┼í╛┬φ┐╦╩2╛▌═°í┐_2019.docx]

各位代表：

　　现在，我代表市人民政府向大会报告工作，请予审议，并请政协各位委员和其他列席人员提出意见。

　　2018年工作回顾

　　过去一年，我们坚持以习近平新时代中国特色社会主义思想为指导，在省委、省政府和市委的正确领导下，全面贯彻落实党的十九大和十九届二中、三中全会精神，深入学习贯彻习近平总书记对广东重要讲话和系列重要指示批示精神，贯彻落实省委十二届历次全会和市委十一届六次全会精神，坚持稳中求进工作总基调，践行新发展理念，落实高质量发展要求，不断解放思想，深化改革开放，统筹做好稳增长、促改革、调结构、惠民生、防风险各项工作，努力开创了全市经济社会发展的新局面。

　　一年来，我们坚持稳中求进，经济保持平稳健康发展

　　面对复杂多变的经济形势，我们坚定发展信心，增强战略定力，保持工作连续性，主动担当作为，推动各项工作稳步向前。预计全市地区生产总值约4150亿元，同比增长6.6%左右（下同）；地方一般公共预算收入可达400亿元，增长2.8%；来源于惠州的财政总收入1188.1亿元，增长18.7%；全体居民人均可支配收入约3.38万元，增长9%。

　　工业经济提质增效。坚持制造业立市，大力发展先进制造业，中海油惠炼二期全面建成投产，大亚湾石化区炼化一体化规模跃居全国第一。TCL模组整机一体化项目一期、旭硝子显示玻璃二期试产，华星光电高世代模组投产。新增年主营业务收入超百亿元工业企业3家，总数达10家。全市规上工业总产值增长8.3%，规上工业企业利税总额增长12%。

　　重大项目加速推进。开展建设项目百日攻坚，集中力量保重点，247宗重点项目投资666亿元，完成年度计划的105%。中海油油品结构升级项目、惠州机场改扩建工程动工建设，大亚湾LNG电厂两台机组建成投运。赣深高铁、广汕汕高铁加快推进，荃湾煤炭码头进港铁路通车。建成武深高速惠州段，新增高速公路98公里，总里程达732公里。建成金恺大道等市政路桥。中韩（惠州）产业园实施方案获省政府批准。

　　市场活力日益增强。优化实体经济发展环境，落实“惠十条”，降低企业成本约202.3亿元。加大工业用地保障力度，全市新增用地指标的71.3%用于工业项目。盘活存量和低效用地1.07万亩。推动市场主体发展壮大，“小升规”企业1100家，其中工业企业485家。5家企业在香港联交所上市或新三板挂牌。新增市场主体约12万个，增长25%，其中企业3.6万家。

　　内外需求平稳增长。大力拓展消费空间，培育城乡消费热点，社会消费品零售总额1477亿元，增长9.4%。限额以上企业网上零售额增长30%。加强对外经贸合作，外贸结构继续优化，进出口总额3314亿元，其中一般贸易出口增长16%。民营经济增加值占GDP比重44%。

　　一年来，我们抓住“窗口期”，乘势而为集聚发展新动能

　　产业项目引进取得重大突破。化危为机、抢抓机遇，引进投资100亿美元的埃克森美孚化工综合体项目，并成功纳入国家石化产业规划布局方案；推动投资70亿美元的中海油/壳牌三期落户大亚湾。全市新备案外资项目1923宗，增长3倍多，合同利用外资金额增长91%。

　　经济结构持续优化。坚定不移调结构，三次产业结构为4.3∶53.1∶42.6。高技术制造业和先进制造业占规上工业增加值比重分别为40.4%、70.6%。现代服务业占比54.9%，提升0.8个百分点。港口吞吐量8757万吨，增长21.4%。惠州机场旅客吞吐量188万人次，增长96.4%。旅游接待人数增长9.3%，旅游收入增长14.2%。农民专业合作社达3020家，获认定首批省级现代农业产业园3家。

　　创新能力不断提升。加大投入支持创新发展，全市R&D经费支出占GDP比重2.3%。新认定高新技术企业435家，总量1108家，增长40%。中科院“两大科学装置”动工建设，潼湖科技小镇首期开园。新增省级高水平新型研发机构3家。规上工业企业研发机构覆盖率46%。PCT专利申请量360件，万人发明专利拥有量13件。“珠江人才计划”创新创业团队增至6个。

　　一年来，我们坚决打好三大攻坚战，加快补齐跨越高质量发展重大关口的短板

　　积极防控化解风险。强化忧患意识、底线思维，出台政府投资项目和财政预算收支三年滚动计划。逐步提高财政收入质量，处理历史欠账30多亿元。制定地方政府债务化解实施方案，严格规范PPP项目管理，化解隐性债务200多亿元。建立商品房项目差别化价格监制机制，房地产市场健康平稳。加强涉众型金融风险排查和化解，推进互联网金融专项整治，地方金融安全稳定。

　　扎实推进脱贫攻坚。完善精准扶贫责任体系，累计投入22.6亿元，实施村扶贫项目2375个，建档立卡有劳动能力的贫困人口人均可支配收入突破1万元，相对贫困人口按年度计划全部实现预脱贫。启动全域推进农村人居环境整治，高标准建设1030个创建项目，46个省定贫困村完成“三清三拆三整治”，达到“干净整洁村”标准。全面推进“四好农村路”建设，惠阳、惠东获评全国示范县区。深入开展“美丽乡村·三大行动”，造林9.89万亩，建成村庄公园177个，完成农村危房改造4960户，农村面貌不断改善。

　　强力推进污染防治。狠抓中央环保督察“回头看”和省环保督察交办案件以及国家海洋督察反馈问题的整改，依法严处一批违法企业，追责问责干部25名。启动供排污一体化改革，系统推动水环境治理。挂牌推进重点流域污染整治，沙河水质由劣V类改善为Ⅳ类，淡水河、潼湖水主要污染物负荷分别下降34.2%、10.2%。建成污水管网406.8公里，改造老旧管网148.5公里。清理整治“散乱污”企业1874家，完成86家省控VOCs企业治理，环境空气质量优良率91.8%，在全国169个重点监测城市中稳居前10位。入列国家水生态文明城市，龙门获评国家生态文明建设示范县。

　　一年来，我们坚持以人民为中心，持续增强群众获得感幸福感安全感

　　社会事业不断进步。坚持教育优先，新增学前教育学位2万个、公办义务教育学位2.5万个，惠州中学如期招生，成功创建省首批现代职业教育综合改革示范市。深化综合医改，建成医联体23个，27家二级及以上公立医院全面取消药品和耗材加成。成功举办首届粤港澳大湾区卫生与健康合作大会。惠州西湖成功创5A，市植物园一期建成开园，东坡祠重修开放。新建村（社区）综合文化服务中心262个，实现行政村（社区）全覆盖。承办全国青少年花样游泳锦标赛等高水平赛事，举办南粤古驿道定向大赛等全民健身活动，一批惠州健儿在国际赛事中取得优异成绩。

　　社会治理效能提升。深入开展扫黑除恶专项斗争，刑事治安警情、“两抢”警情分别下降23%、81.4%，实现176天“两抢”零发案，全民禁毒重点工作综合考评全省第一。建成市级公共法律服务中心，公共法律服务平台实现四级全覆盖。铁腕整治“两违”，查处违法用地5175亩，拆除违法建筑359万平方米。省级食品安全示范城市创建深入推进，有效保障群众饮食用药安全。深刻汲取“8·1”交通事故教训，超常力度治超，生产安全事故死亡人数下降14.7%。支持群团改革，更好发挥工青妇联系群众桥梁纽带作用。协助驻惠部队做好停偿工作，双拥共建不断巩固。

　　社会保障扩面提标。多措并举推动就业，新增就业人员7.2万人，失业再就业1.8万人，城镇登记失业率2.3%。城乡居民医保补助标准提高到每人每年490元，39家医疗机构实现全国医保异地就医结算。实施困难群众二次医疗救助，每人每年最高5万元。开展全民预防保健，为127万常住人口免费健康体检。支持发展公益事业，充分发挥红十字会、慈善机构、社会组织在扶贫济困中的积极作用。城乡居民低保标准提高到每人每月800元，城镇、农村特困人员供养标准分别提高到每人每月1750元、1280元。

　　成功防御“8·30”特大暴雨和强台风“山竹”。面对突破历史极值的持续强降雨和罕见风暴，提前转移安置群众，精准调度水库运行，科学组织抢险救援，全市人民众志成城防洪抗灾，实现了“零死亡”。这两场气候灾害防御战，充分展现了患难与共、风雨同舟的军民深情，充分展现了各级各部门高效协同、团结奋战的强大合力，这是惠州砥砺奋进、铿锵前行的强大正能量！

　　一年来，我们坚持以习近平总书记对广东系列重要指示精神统领各项工作，确保了政府工作的正确方向。我们把深入学习贯彻党的十九大精神和总书记重要讲话精神作为头等大事和首要政治任务，在学懂弄通做实上狠下功夫，自觉在思想上政治上行动上同以习近平同志为核心的党中央保持高度一致。把全面从严治党贯穿政府工作全过程，扎实开展“不忘初心、牢记使命”主题教育，勤勉尽责，依法行政，廉洁用权，加快转变政府职能。服务型政府建设持续推进，积极开展“减证便民”行动，取消证明事项53项，取消行政职权60项，下放（委托）县区实施33项。推进“集成办理、一次搞定”政务改革，新网上办事大厅顺利上线。首席服务官、“中介”超市等改革经验全省推广，入选全国首批社会信用体系建设示范城市。法治政府加快建设，坚持向市人大及人大常委会报告工作，向市政协通报情况，共办理市人大议案8件、省市人大代表建议167件、省市政协提案325件。提请市人大常委会审议法规草案1件，全面清理市政府规范性文件746件。大力推进政务公开，发布政府信息18.9万条。廉洁政府建设取得新成效，认真落实党风廉政建设责任制，全力抓好中央、省委巡视及国务院大督查反馈问题整改，严格执行中央八项规定，全市性政府会议压减32%、政务公文减少30%，严格规范公务接待，加大审计监督力度，正风肃纪的常态化机制逐步形成。

　　过去一年，我们积极应对各种挑战，赢得了发展的主动，“稳”的基础更加牢固，“转”的步伐更加坚定，“进”的态势更加积极。这些成绩是市委正确领导、科学决策的结果，是市人大市政协有效监督、大力支持的结果，是全市广大干部群众团结奋进抓发展、抓稳定，一步一个脚印实干出来的。在此，我代表市人民政府，向全市人民、驻惠部队官兵、各民主党派、人民团体以及港澳台同胞、社会各界人士，表示崇高的敬意和衷心的感谢！

　　回顾一年来的工作，对照市十二届人大三次会议确定的工作目标，我们总体上较好完成了全年任务，但地区生产总值、固定资产投资、进出口总额等一些经济指标增幅与预期有距离。这既有宏观经济整体下行因素的影响，也反映出我们工作还存在很多不足。我们清醒地认识到，发展不充分仍是惠州最大的实际，总量不够大、企业不够多、百姓不够富是客观现实，立足客观条件开展工作做得不够好。制约高质量发展的体制机制障碍依然存在，营商环境有待优化，创新体系有待完善。城乡区域不协调不平衡的问题比较突出，中心城区和山区县、城市和农村在经济发展、收入水平、公共服务等方面差距仍然较大。实体经济面临困难较多，用工难、融资难、办事难等问题有待化解。民生事业发展与群众期盼还有差距，入托、上学、看病、出行仍是群众普遍关注的热点问题。

　　各位代表！在改革开放40周年的重要时刻，习近平总书记亲临广东视察，对广东发展提出了明确要求，为我们指明了前进方向、提供了根本遵循。省委充分肯定惠州的工作思路和发展目标，为惠州新一轮改革发展增强了奋进动力、提供了科学方法。我们一定牢记嘱托、解放思想、实干担当，紧紧依靠全市人民，敢于直面问题、解决问题，把发展的眼光放得更远，发展的步子迈得更大，发展的质量提得更高，决不辜负全市人民的美好期望！

　　2019年工作安排

　　2019年，是新中国成立70周年，是全面建成小康社会关键之年，对惠州建设国内一流城市至关重要。当前，受中美贸易摩擦等不确定因素影响，经济下行压力仍然较大，但我们也拥有难得的发展机遇。国家大力推进粤港澳大湾区建设，惠州独具区位、空间、生态等优势，有利于各类资源要素向惠州集聚。省委提出构建“一核一带一区”区域发展新格局，我市的发展重心处于全省沿海经济带主战场上，有利于形成经济新增长极。我们重点发展的石化能源新材料和电子信息产业集群，符合新一轮世界科技革命和产业变革发展方向，将有力促进产业加速迈向中高端、提升竞争力。我们成功引进一批令人振奋的百亿美元级龙头项目，正在推动建设的50亿元以上重大项目有22宗，新一轮发展蓄势待发。我们要抢抓机遇，切实把习近平总书记视察广东重要讲话精神、省委对惠州提出的目标要求转化为惠州发展的生动实践，以只争朝夕的拼搏精神、一往无前的奋斗姿态，全面掀起干事创业新热潮。

　　今年政府工作总体要求是：高举习近平新时代中国特色社会主义思想伟大旗帜，全面贯彻党的十九大、十九届二中、三中全会和中央经济工作会议精神，全面贯彻落实习近平总书记对广东重要讲话和对广东工作一系列重要指示精神，贯彻落实省委十二届历次全会精神，按照市党代会和市委全会的部署要求，坚持稳中求进工作总基调，坚持新发展理念，坚持推进高质量发展，坚持以供给侧结构性改革为主线，坚持深化市场化改革、扩大高水平开放，扭住建设粤港澳大湾区这个“纲”，统筹推进稳增长、促改革、调结构、惠民生、防风险工作，进一步稳就业、稳金融、稳外贸、稳外资、稳投资、稳预期，提振市场信心，继续打好三大攻坚战，着力提高发展平衡性和协调性，保持经济持续健康发展和社会大局稳定，在打造珠江东岸新增长极、粤港澳大湾区高质量发展重要地区和国内一流城市的新征程上迈出坚实步伐，为全面建成小康社会收官打下决定性基础。

　　今年经济社会发展的主要预期目标是：

　　国内生产总值增长7%，规上工业增加值增长7.5%；

　　一般公共预算收入增长4%，税收收入占比75%以上；

　　固定资产投资增长12%，重点项目年度投资计划688亿元；

　　社会消费品零售总额增长9%；

　　进出口总额保持正增长，一般贸易出口占比30%以上；

　　R&D经费支出占GDP比重2.4%；万人发明专利拥有量14件；

　　居民人均可支配收入增长8%；城镇登记失业率2.6%以内；

　　环境空气质量优良率保持90%以上；饮用水源水质达标率保持100%；主要污染物排放、单位GDP能耗等指标完成省定目标。

　　建设国内一流城市，实现上述年度目标，要全面落实市第十一次党代会第四次会议精神，推动思想再解放、改革再提速，因地制宜，创造性抓好落实，处理好稳和进的辩证关系。突出“稳”，以稳求进。坚决把“六个稳”要求落到实处，坚持底线思维，抓实风险防控、民生保障、社会稳定等工作。稳不是无所作为，不是机械求稳，需要我们凝心聚力办好惠州自己的事，确保经济运行在合理区间。强化“进”，以进促稳。树立抢抓机遇意识，大力实施逆周期操作，更好发挥投资的关键作用、消费的基础作用，着力优化营商环境，进一步激发微观主体活力，发展壮大实体经济。继续实施大项目带动战略，以沿海经济带为发展主战场，加快建设大交通、大产业、大平台，推动经济高质量发展。

　　重点抓好以下七方面工作：

　　（一）集中力量打造“2+1”产业集群

　　一流城市必须具备一流的实力，实力来源于强劲的产业支撑。我们要打破零敲碎打、承接低端产业转移的传统模式，走好产业集群发展的路子，聚焦规模效应综合优势，集中力量用8-10年时间打造石化能源新材料、电子信息两大万亿级产业集群，加快发展生命健康产业。

　　打造世界级绿色石化产业基地。抓住国家调整石化产业规划布局机遇，积极推进埃克森美孚化工综合体、中海油/壳牌三期等引领性项目，加快项目报批、三通一平等前期工作，促进项目尽早开工建设。充分发挥大项目引领作用，加快推进中海壳牌SMPO/POD、中海油油品结构升级项目等建设。做精做深产业链，重点发展高端化学品、聚酯新材料和生物材料。对标世界一流石化园区，重整提升大亚湾石化区基础设施，全面升级改造道路交通、码头航道、安防通讯等设施，谋划建设具有国际水平的石化技术研发中心。拓展产业发展空间，在惠东白花谋划新材料园区，加快编制园区规划，启动专用通道、排洪排污管廊论证等前期工作。

　　做强国家电子信息产业基地。加快珠三角（惠州）国家自主创新示范区建设，壮大电子信息产业集群。大力发展超高清视频产业，推动旭硝子显示玻璃二期、液晶模组整机一体化一期投产。扩大4K电视试点示范城市建设范围，全市4K用户数达60万户以上。提前布局5G产业，鼓励企业研发消费类5G智能终端和配件，推动龙旗二期、光弘二期等项目竣工投产。促进互联网、大数据、人工智能与制造业深度融合，培育工业机器人产业，做优汽车电子产业。规划建设仲恺人工智能产业园、惠阳金融科技城，推动中电科技等项目开工。

　　建设全省中医事业创新示范城。编制实施健康产业发展规划，扶持中医药产业发展。抓好中医科学大会成果转化，引进高技术生物制药企业和研发机构。鼓励山区因地制宜种植中药材，建设岭南中药材种植园，发展药材药品交易市场。引导社会资本建设高水平医养结合康复机构，推进罗浮山医养项目、葛洪中医院建设。促进“健康+”旅游、体育、文化、农业、林业等产业融合发展，打造龙门森林温泉养生、惠东滨海休闲度假等康养基地。

　　推动“2+1”产业集群发展，要同步发展生产性服务业，大力发展民营经济，强化要素保障和政策扶持。探索建设电子信息及化工产品交易平台，大力发展现代物流、供应链服务等现代服务业，力争生产性服务业占比提升1个百分点。强化金融支持，推动设立“2+1”产业集群发展基金，推广“简单汇”等供应链融资模式。拓宽融资渠道，支持鼓励企业上市。强化工业项目用地保障，继续保持市县区年度工业用地供给比例不低于50%。出台“民营经济十条”，落实减税降费政策措施，扶持中小微企业做优做强，培育更多“专精特新”隐形冠军，新增规上工业企业200家以上。

　　打造万亿级产业集群是一项宏大的系统工程，既要有心无旁骛的专注，久久为功，一年接着一年干；更要有看准就干的果敢，雷厉风行、勇往直前，才能在激烈的市场竞争中，赢得主动、率先发展。

　　（二）打造高水平开放创新大平台

　　一流城市必然是充满活力的开放创新城市。我们要高起点建设开放创新平台和载体，增强创新资源集聚能力和创新成果转化能力，加快形成创新创业活力最强的开放新高地和发展引擎，成为粤港澳大湾区高质量发展重要地区。

　　高标准建设中韩（惠州）产业园。大力营造国际化营商环境，优先复制自贸区贸易、投资、金融、人员流动便利化等创新经验。加快推动起步区形成集聚发展的良好态势，抓好智慧大道主干路网、市政管网、供水供电等基础设施建设，推动赛格潼湖半导体产业基地、SKI-亿纬锂能动力电池等一批项目动工建设。密切加强与韩国等发达国家和地区的合作，促进科技交流、产业对接、资源共享。理顺管理体制机制，全面启动产业园六大片区建设，用足用好政策，加快项目引进落地，把中韩（惠州）产业园打造为各县区开放创新的重要阵地。

　　高水平推动能源科技岛建设。科学编制发展规划，把稔平半岛建设成为大湾区能源基地和科技创新中心。加快港口海上风电项目建设，做好LNG接收站前期工作，发挥隔墙供应优势，布局建设天然气电厂、大湾区数据中心等项目。依托中科院“两大科学装置”，整合全市多种能源产业基础和创新资源，谋划建设现代物理与能源省实验室。规划建设能源环保产业园，发展能源装备制造业。

　　加快集聚创新资源。积极对接广深港澳科技创新走廊，面向港澳引进国际领先技术，推动科技金融、科技服务等领域合作；面向深圳引进科技型企业、风险投资机构，促进科技成果在惠转化；面向广州引进高校、科研院所资源，吸引更多高端人才到惠创业就业。要强化企业创新主体地位，着力解决人才落户、住房、子女入学等问题，推动创新人才向企业集聚。支持企业与高校、科研院所共建研发机构，组建市级以上工程技术研发中心50家，力争规上工业企业设立研发机构比例达48%以上。培育一批成长型科技企业，全市高新技术企业总数超过1300家。

　　优化外资外贸结构。积极参与“一带一路”建设，联合港澳等大湾区城市，携手合作引进来，“拼船出海”走出去。强化产业链上下游延伸，吸引更多世界500强和行业龙头企业在惠设立总部、生产基地。紧盯日韩、欧洲重点地区，引进一批高端电子信息领军企业。落实省、市“外资十条”，大力推动备案外资项目落地。全力保持外贸稳定，加快创建服务外包示范区、电子商务服务外包产业园，支持外贸综合服务企业发展，帮扶企业开拓国际市场。加快海关特殊监管区建设，先行申报B型保税物流中心。着眼于更好满足消费需求变化和产业转型需要，鼓励扩大高水平进口，重点支持新型平板显示、新能源等先进技术设备及零部件进口。

　　开放增活力，创新强动力。我们要以更加开放的眼光、包容四海的胸怀，努力营造公平竞争、优胜劣汰的发展环境，让各类人才施展抱负、实现梦想，让各类企业同台竞技、创新争先，让惠州大地再现“孔雀东南飞”的盛景！

　　（三）建设令人向往的现代化品质城市

　　一流城市要有卓越的特质，彰显悠久的历史、独有的魅力。我们要以国际国内先进城市为标杆，统筹社会与自然资源，在城市规划建设和精细化管理等方面下大功夫，提升城市品质和美誉度，更好满足广大群众对美好生活的期待。

　　全面融入“一核一带一区”区域发展新格局。顺应粤港澳大湾区城市群建设和全省区域发展新形势，着力推动惠城、惠阳、大亚湾、仲恺等核心片区优化发展，加快惠东、博罗重点拓展区发展步伐，提升两大片区能级，整体融入“一核”；推动惠东、惠阳、大亚湾沿海地区联动发展，打造现代化沿海经济带，全面对接“一带”；推动龙门、博罗北部、惠东北部绿色发展，筑牢生态屏障，助力构筑“一区”。强化规划引领和管控作用，深入研究城市形态和发展方向，科学划定中心城区增长边界和承载极限，推动城市精明增长、集约发展。加快国土空间规划试点工作，推动“多规合一”，完成全市土地利用总体规划调整，统筹配置好全市土地资源。

　　构建融入大湾区的大交通体系。加快对外大连通，建设半小时通达广深港特大城市的快速通道，抓紧对接莲塘口岸等骨干工程，全面推进赣深高铁、广汕汕高铁建设，上半年开工建设惠州北站、惠城南站等高铁枢纽站场。推进千万级新干线机场建设，建成惠州机场新航站楼，力争开通国际口岸。加强与周边城市高速公路互联互通，建成从莞高速、惠清高速，加快河惠莞高速建设，开工建设惠龙高速、深汕西高速改扩建工程。加快中心城区交通大循环建设，春节前建成仲恺大道，年底前建成小金河大道等5条市政路，推进金龙大道等快速化改造和安全提升，力争通过3年左右时间，形成“两环八射”主干路网，实现中心城区15分钟上高速。

　　加快智慧城市建设。按照“信息畅达、万物互联”的理念，推动信息化基础设施升级扩容，整合完善城市基础数据库，促进信息开放共享，构建“智慧城市大脑”。建设“数字政府”，推动一网通办，支撑更多事项“掌上办”，实现政务服务全天候运作、便捷高效。完善“智慧市政”，实时感知、管控水电气等市政基础设施。升级“智慧城管”，加快城乡管理资源网格化整合，促进管理精细化。推进“智慧环保”，实现环境违法行为及时发现、有效取证、依法解决。优化“智慧警务”，提升打击防范、应急处置、服务群众的智能化水平。发展“智慧交通”，建成综合交通网络运行指挥中心。全力疏通市民反映强烈的22个堵点，年内完成一半以上。推进1.6万个公共停车位智能化管理，开工建设5座以上公共智能停车楼，鼓励开放共享私有停车位，缓解市区停车难问题。

　　大力推进城市形象革新。加强城市设计，强化城市空间、天际线、色彩、建筑立面管控和引导，塑造公园城市特色风貌。树立精品意识，严格落实建设工程质量强制标准和质量法规，以工匠精神打造百年建筑。落实好住宅小区开放管理要求，鼓励已建成住宅小区“拆挡见绿”，实现内部景观公共化。开展城市园林、街景整治，提升城市出入口和重要交通节点环境品质，打造独具特色的靓丽风景线。科学规划“三旧”改造单元，连片推动城市更新，完成改造5000亩以上。以“绣花功夫”改造升级老旧社区、城中村，积极推进背街小巷“微改造”和城郊结合部综合整治。抓好水东街等5条历史文化街区升级改造和历史建筑保护利用，更好延续历史文脉，让惠州这座国家历史文化名城焕发新活力。

　　人们为了生活来到城市，为了生活得更好而留在城市。一流城市不仅要有高水平的硬件设施，也要有精细化的城市管理，更要有高度自觉的文明行为，需要全民参与、人人出力，共筑美丽智慧和谐家园。

　　（四）厚植绿水青山生态优势

　　一流城市要有优质的生态环境。我们要坚定不移推进绿色发展，坚持污染防治与生态修复并举，源头治理与集中攻坚并重，严守生态保护红线、环境质量底线、资源利用上线，让良好的生态成为高质量发展的重要支撑。

　　打好污染防治攻坚战。系统推进水环境综合治理，完成河湖“五清”专项整治，集中剿灭劣V类河涌，集中力量打好淡水河整治翻身仗，巩固提升沙河、潼湖水、公庄河水质。建成5座污水处理厂，完成43座污水处理厂提标升级，新增日处理能力25万吨。新建排水管网130公里，截污管网337公里。完成27条城市黑臭水体整治主体工程，实现国考、省考断面水质持续改善，全市地表水优良比例77.8%以上，确保东江水质稳定优良。完善空气质量监管网络，抓好压减燃煤、移动源污染和扬尘治理，全面完成“散乱污”企业综合整治，确保环境空气质量稳定优良。开展重点行业用地土壤污染状况调查，稳步推进污染地块修复。

　　强化固废污染防治。提升固废处置能力，建立健全全过程监管体系。建成市生态环境园一期，推进市区生活垃圾焚烧发电厂技术改造，加快建设市生态环境园二期、榄子垅环境园二期等项目，新建、升级改造一批垃圾转运站，确保城市生活垃圾得到100%有效处置。建成东江威立雅二期危废处置项目，大力推进水泥窑协同处置固废危废项目建设，推动建筑垃圾处理设施县区全覆盖，实现全市固废危废、城市生活污泥处理处置率100%。加强沟通协作，严厉打击非法转移倾倒固废行为。

　　持续加强生态修复。坚持保护优先、自然恢复，实施湿地修复工程，推进潼湖国家湿地公园（试点）建设，新建惠东好招楼海洋湿地公园。实施水系连通工程，抓好金山湖、小金河等流域水环境生态修复综合整治。实施岸线修复工程，建设生态堤岸、美丽海湾，打造千里碧道。实施绿道提升工程，不断完善绿道线网。实施森林公园建设工程，推动象头山、九龙峰创建国家级森林公园。植树造林1.4万亩，改造残次林、低效林2.8万亩。

　　良好的生态、优美的环境，是惠州的“金字招牌”。我们在上大项目时要采用最高的技术标准，实施最严的环保要求，做好最周全的防控措施，坚决守住生态底线，让蓝天碧水青山绿岸成为惠州最鲜明的城市特色和发展优势。

　　（五）全面实施乡村振兴战略

　　建设一流城市，最艰巨最繁重的任务在农村。我们要围绕“五大振兴”，破解城乡二元结构，把短板变成“潜力板”，促进城乡协调发展，提高一流城市成色。

　　打赢精准脱贫攻坚战。全面落实“一户多策、一人一策”精准要求，重点推进就业扶贫、产业扶贫、教育扶贫，充分发挥农民主体作用，激发群众致富奔康内生动力。巩固脱贫成果，完善扶贫大数据平台，大力实施兜底保障工程，做到脱贫不脱保，确保全市建档立卡的37895贫困人口稳定脱贫，真脱贫、不返贫。整体推进示范片规划建设，完成46个省定贫困村创建示范村工作，年人均可支配收入达到全省农民人均可支配收入的60%。102个市级相对薄弱村优先启动美丽宜居乡村建设，补齐基础设施、公共服务短板。通过示范村产业辐射带动、基础设施延伸连通，以点带面促进周边农村发展。

　　繁荣乡村产业。推动一二三产业融合发展，积极引导加工、物流、商贸等企业进乡村，推进一批现代农业产业园建设。发展休闲观光农业，打造一批农业公园。培育家庭农场、农民合作社等新型经营主体，促进小农户与现代农业发展有机衔接。因地制宜布局农业产业功能区，做优做强丝苗米、马铃薯、梅菜等特色品牌，支持发展农产品精深加工，提升农产品附加值，新增农业龙头企业3家以上。发展农村电商，强化城乡产销对接，以城市大市场带动农民持续增收。实施新型职业农民培育工程、“东江菜师傅”工程，培养农村实用型人才。

　　实施“百村示范、千村整治”工程。推动村庄建设规划全覆盖，落实“一户一宅”管理要求和农房风貌管控指引，鼓励农房风貌改造。广泛发动群众参与人居环境整治，全面完成“三清三拆三整治”任务，推进农村“厕所革命”，80%以上村庄要完成环境综合整治。加快示范村创建，80%以上行政村达到“干净整洁村”标准。继续推进“四好农村路”建设，完成窄路拓宽623公里、村道路面硬化236公里。实施农村饮水水质提升工程，确保群众饮水安全。开展文明村镇、文明家庭创建，80%村镇达到省文明村镇创建标准，焕发乡风文明新气象。

　　进一步提升水利防灾减灾能力。立足防大汛、防大涝、防大灾，抓好防洪排涝设施建设，加快白盆珠等水库除险加固工程和观洞水库应急备用水源工程等项目建设。加快惠东白花河、博罗公庄河、龙门蓝田河等中小河流治理，完成河道疏浚、整治100公里以上。建设智慧水文监测系统，提升水情水文预测预警能力。完善农村水利基础设施，实施一批农田高效节水灌溉和小水库、小山塘、小灌渠改造工程。

　　加快发展全域旅游。依托丰富的历史文化资源和优美的生态环境，推动文旅、农旅融合发展。支持博罗、龙门创建国家全域旅游示范区，承办好2019广东旅游文化节。抓好“环罗浮山—南昆山”生态旅游区、“大亚湾—稔平半岛”滨海旅游产业带、东江西枝江滨江风景道建设，规划建设滨海旅游公路。完善旅游集散咨询、智慧旅游、旅游厕所等服务设施，提升旅游服务水平。加强传统村落、历史建筑、革命遗址、南粤古驿道修复保护和活化利用，打造精品乡村旅游线路，规范发展乡村民宿，壮大乡村旅游业。

　　实施乡村振兴战略是一篇大文章。我们要遵循乡村发展规律，不搞一刀切，不搞大拆大建，注重地域特色，体现乡土风情，让乡村成为城里人留恋、村里人乐居的幸福家园。

　　（六）擦亮惠民之州品牌

　　建设一流城市的根本目的是富民惠民，让改革发展成果更多更公平惠及全市人民。我们要突出保基本兜底线，确保民生支出只增不减，解决好群众普遍关心的教育、医疗、养老等问题，不断提升共建共治共享水平，实现经济发展与民生福祉互促共进。

　　推动教育均衡优质发展。公平配置教育资源，推动学前教育、义务教育“齐步走”，落实省学前教育、公办普通高中生均经费拨款制度。全面实施“县管校聘”制度，每年义务教育阶段教师交流人数占比不低于5%。鼓励优质学校集团化办学，促进优质教育资源向农村和相对薄弱学校流动。加快小区配套幼儿园、农村幼儿园建设，确保常住人口4000人以上行政村单独办园全覆盖，全市新增学前教育学位2万个以上。加大中小学校建设力度，新增义务教育学位2.4万个。围绕产业发展和就业需求，提升职业院校、技工院校办学质量，培育更多产业技能人才。大力推动省市共建惠州学院，支持电子、化工、材料、能源、师范等重点特色学科建设，打造根植惠州、服务大湾区的高水平应用型大学。发展社区教育、终身教育，举办院士专家大讲堂，提升市民科学素养。

　　丰富群众文体生活。深入挖掘惠州历史名人文化、红色文化、客家文化内涵，全面展示历史文化名城的深厚底蕴。办好第10届东坡文化节，建成东坡纪念馆。推进高潭“三大基地”建设，建成省港大营救历史陈列馆。建设市美术馆、非物质文化遗产展示中心等文化项目，筹建岭东雄郡文化创意产业园。推广图书馆、文化馆总分馆制，提升公共文化服务效能。积极发展文化产业，开展百场文化惠民活动，推出一批具有惠州特色的文艺精品，提升城乡居民文化素养。大力发展体育事业，广泛开展全民健身活动。

　　推动医疗卫生资源下沉。按照“大病不出县、小病不出镇”要求，全面加强县区综合医疗能力建设，推动8家镇卫生院改造升级为县区“第二人民医院”，完成惠东多祝、博罗杨村卫生院升级改造，全面完成村卫生站标准化建设，实现县域内住院率超75%、基层医疗卫生机构门诊量占比65%以上。加强卫生人才引进培养，引导推动高水平医务人员往基层走，每万人口全科医生数达3名。深入推进“三医联动”，重点抓好药品跨区域联合集中采购、现代医院管理制度示范点、医联体建设等工作。推进高水平专科建设，创建3个省级水平临床重点专科，支持市中心人民医院争取进入全省高水平医院“登峰计划”建设范围。鼓励社会资本参与高端医疗机构建设，大力发展精准医疗，扩大优质医疗资源供给。

　　促进长者乐享善为。充分发挥长者智慧资源，支持长者利用知识、经验和技能优势，积极参与社会公益、咨询服务、社区治理、乡村发展，鼓励退休医生、教师到基层支医支教。提升养老设施标准和管理水平，推进乡镇敬老院升级改造。推进居家养老信息化服务平台建设，支持社会工作者为长者提供专业化服务，开展老年人家庭医生签约服务，逐步建立农村留守老年人巡访制度。在有条件的社区建设长者日间照料中心、开设长者饭堂，探索开展老年人“大配餐”试点。将老年人意外伤害综合保险扩面至户籍60周岁以上，实现政府统保全覆盖。筹备设立长者服务专门机构，推动老年人事业高水平发展。

　　实施增收致富工程。做强富民经济，支持民营企业发展，创造更多就业岗位。抓好高校毕业生多渠道就业，强化重点群体职业技能培训，促进就业困难人员再就业3000人以上，新转移农村富余劳动力8000人，新增城镇就业6.5万人。着力增加城乡居民经营性收入，落实好国家和省促进创业就业政策，将一次性创业资助标准从5000元提高至1万元，资助范围扩大到返乡创业人员。多渠道增加居民财产性收入，积极培育专业理财、资产管理等服务机构，发展普惠金融和房屋租赁市场。

　　提高基层治理水平。推动治理重心下移，将镇街工作重心从经济管理转向社会治理，完善“综治中心+网格化+信息化”治理模式，以社区村落为基本单元引导居民自我管理，把问题解决在基层、矛盾化解在基层。纵深推进扫黑除恶专项斗争，持续深化全民禁毒工程。深入开展全民普法行动，加强基层公共法律服务。推动基层“三社”联动，逐步实现社区社会工作全覆盖。落实安全生产责任制，突出抓好石化行业、交通领域安全生产，开展事故多发路段和安全隐患点专项治理。整合应急救援资源，提高快速反应和处置能力。加强食品安全专项整治，确保群众“舌尖上的安全”。完善地方金融风险监测预警平台，确保不发生区域性系统性金融风险。坚持“房住不炒”定位，推动房地产市场健康平稳发展。依法保障妇女、儿童权益，支持工会、妇联在化解社会矛盾中有更大作为，深入开展双拥共建，做好对口援建，推动各项事业全面发展。

　　民生无小事。我们要真心为民办事，从小处着眼、实处入手，尽力而为、量力而行，多雪中送炭、少锦上添花、不做“盆景”,切实解决百姓的难事烦事，真正让民生工程成为民心工程。

　　（七）实施引领发展的战略性战役性改革

　　一流城市要有与之相适应的现代治理体系。当前，改革正处在“船到中流浪更急、人到半山路更陡”的攻坚期和深水区。我们要坚定推动改革提速，瞄准国内外最优最好最先进，聚力打造一流营商环境，形成管理科学、运行高效的体制机制。

　　要建立健全县区差异化高质量发展机制。按照市委要求，落实以功能区为引领的区域发展新战略，针对每个县区“量体裁衣”，实施各有侧重、各有特色的“1+7+1”一域一策评价体系。体现高质量发展要求，引导各地探索符合实际的灵活的发展道路，着力提升城市能级、产业发展、环境质量、乡村振兴、营商环境、公共服务等方面水平。充分发挥各县区比较优势，让每个功能区由原来单一发展模式的“跟随者”转变为各自功能区的“引领者”，让各县区各尽其能、各展所长，又协同配合、相互促进。

　　对标自贸区优化营商环境。深化“放管服”改革，打造“比深圳快一步”的行政效率。压缩企业开办环节，将企业开办时间压缩至5个工作日以内。完善“多证合一”“证照分离”机制，建设以信用为核心的法治化市场监管体系，破解企业“准入不准营”问题。建立投资项目事前咨询服务制度，全面推行报建承诺制和否决申诉救济制度，促进项目高效落地。打造企业疑难事务快办平台，落实领导约访接访制度，开通解决企业困难“直通车”。加强经常性规范化政企沟通，建立以企业感受度为导向的政策评估机制，使服务企业更贴心、更到位。整合12345“惠民意”统一服务热线，实现建议有渠道、咨询有人答、办事有人理、投诉有平台。

　　健全集中力量保重点机制。坚持领导带头抓落实，各级领导干部要履职尽责、一抓到底，全面统筹重点项目的督导推进、落地保障工作。用地指标优先保障重点项目，建立用地报批绿色通道。积极拓展融资渠道，争取更多专项债券集中用于重点项目，优先支持重点项目企业发行债券、短期融资券和中期票据。完善城市建设投融资机制，更好发挥市城投、交投、水投等国企的主力军作用，引导民间资本参与城市基础设施建设。完善要素价格监管机制，做好砂石、水泥、水电、用工等要素保障，确保重点项目加快建设、早见成效。

　　优化城乡公共管理机制。加快供排污一体化改革，推动供排污设施投资建设、运营管理市场化，系统推进全市域水环境治理。实施固体废物管理体制改革，打破行政区划界限，统筹布局全市固废处理设施，构建就近处置、辐射周边的处置处理体系。推动环卫一体化改革，整合环卫力量，“一把扫帚扫到底”，实现城市保洁一家管、全覆盖。深化综合交通运输改革，推动城市道路、公路、水路、铁路行业管理职能合一，实现“交通一张网”管理。完善质量管理体系，整合业务相近的检验、检测、认证机构，提升公共检测服务效能。实施城乡管理综合行政执法改革，形成一体高效的行政执法体系，消除城乡建设管理盲区。

　　市委十一届七次全会通过了惠州市党政机构改革方案。我们要坚决落实好机构改革要求，改出执行力、创新力，加快转变政府职能，构建优化协同高效的机构职能体系，更好牵引、支撑和保障各领域改革。

　　推动高质量发展，建设国内一流城市，对政府能力和作风提出了新的更高要求。我们一定牢固树立“四个意识”，坚定“四个自信”，坚决做到“两个维护”，在思想上政治上行动上同以习近平同志为核心的党中央保持高度一致。全面贯彻落实中央决策部署和省委省政府工作安排，坚持在市委领导下开展工作，自觉接受人大监督、政协民主监督，持续推进思想再解放，不断提高行政效能，大力提振干事创业精气神，推动改革再提速、工作再落实。

　　各位代表！新的一年，形势复杂多变，危机中孕育着机遇。强者总是善于危中取机、变中求胜，最终脱颖而出、引领发展。新时代是奋斗者的时代，机遇总是垂青勇于竞争的人。我们一定更加紧密团结在以习近平同志为核心的党中央周围，以习近平新时代中国特色社会主义思想为指导，在省委、省政府和市委的坚强领导下，不忘初心、牢记使命，闻鸡起舞、日夜兼程、风雨无阻，努力把惠州打造成为珠江东岸新增长极、粤港澳大湾区高质量发展重要地区和国内一流城市，向全市人民交出合格答卷！
